# A randomized controlled trial on the effects induced by robot-assisted and usual-care rehabilitation on upper limb muscle synergies in post-stroke subjects

**DOI:** 10.1038/s41598-021-84536-8

**Published:** 2021-03-05

**Authors:** T. Lencioni, L. Fornia, T. Bowman, A. Marzegan, A. Caronni, A. Turolla, J. Jonsdottir, I. Carpinella, M. Ferrarin

**Affiliations:** 1 IRCCS Fondazione Don Carlo Gnocchi, Milan, Italy; 2 Department of Medical Biotechnologies and Translational Medicine, Università Degli Studi di Milano, Milan, Italy; 3 Movement Neuroscience Research Group, IRCCS San Camillo Hospital, Venezia, Lido Italy

**Keywords:** Rehabilitation, Stroke, Motor control

## Abstract

Muscle synergies are hypothesized to reflect connections among motoneurons in the spinal cord activated by central commands and sensory feedback. Robotic rehabilitation of upper limb in post-stroke subjects has shown promising results in terms of improvement of arm function and motor control achieved by reassembling muscle synergies into a set more similar to that of healthy people. However, in stroke survivors the potentially neurophysiological changes induced by robot-mediated learning versus usual care have not yet been investigated. We quantified upper limb motor deficits and the changes induced by rehabilitation in 32 post-stroke subjects through the movement analysis of two virtual untrained tasks of object placing and pronation. The sample analyzed in this study is part of a larger bi-center study and included all subjects who underwent kinematic analysis and were randomized into robot and usual care groups. Post-stroke subjects who followed robotic rehabilitation showed larger improvements in axial-to-proximal muscle synergies with respect to those who underwent usual care. This was associated to a significant improvement of the proximal kinematics. Both treatments had negative effects in muscle synergies controlling the distal district. This study supports the definition of new rehabilitative treatments for improving the neurophysiological recovery after stroke.

## Introduction

Stroke is among the most frequent causes of adult-onset disability^1^, requiring a compelling medical and social need for rehabilitation. Among the most disabling post-stroke impairments are those affecting the contralesional upper limb, which include loss of movement, coordination, sensation, and dexterity. Even though substantial research efforts have been devoted to improve functional recovery^2^, motor rehabilitation in the upper extremity is still a challenging issue because of the limited understanding of the neurophysiological mechanisms underpinning motor recovery and the lack of interventions with demonstrated long term effectiveness^2^.

Since one of the primary goal of rehabilitation is to make patients independent, very often the training performed immediately after the stroke is focused on the recovery of walking. However, arm skills are also fundamental not only for activities that require fine movements such as grasping, manipulation, functional use of objects, but also for global abilities such as walking and balance reactions^3,4^. Furthermore, the non-recovery of the upper limb, which is often persistent, causes disabling conditions and is a major contributor to the reduced quality of life^5,6^.

The recovery after a stroke depends on a large repertoire of functional and structural processes within the central nervous system (CNS), named neuroplasticity, which may occur spontaneously but can also be induced by movement practice^7^.

Robot-assisted arm training has shown promising results for improving activities of daily living (ADLs), arm function, and arm muscle strength after stroke^8,9^. Randomized control trials (RCTs) have been carried out to clarify if robot-assisted therapy is able to produce better effects compared to usual care in terms of motor function improvement of the upper limb. The results suggest that these treatments show similar effectiveness in improving upper limb motor performance, as measured through clinical scales^10,11^. However, it should be noted that an instrumented analysis of upper limb movements during a functional task has recently shown that robot therapy induces larger improvements of shoulder/elbow coordination and greater reduction of compensatory movements than usual care treatments^11^. Overall, there are evidences that intensive, repetitive and functional motor exercises assist recovery and rehabilitation^12^. For this reason, robotic devices have been introduced in the rehabilitation field as tools to facilitate repetitive practice of limb movement, specifically in the upper extremity. The added value of robotic devices that support and guide the subject during the movement lays in the possibility of restoring neurophysiological pathways that are as much as possible similar to those of healthy subjects^13–15^. However, there is poor understanding of robot-induced motor learning in the CNS^16^. In addition, a still open question is whether the application of motor learning principles can enhance the transfer of planar robot-assisted rehabilitation effects also to non-trained 3D motor tasks, typical of ADLs^17,18^, that involve both proximal and distal parts of the upper limb.

The evaluation of behavioral parameters together with the measure of neurophysiological signals, such as the electromyographic activity (EMG), opens the possibility for a comprehensive characterization of motor control and consequently of its recovery after a neurological injury, providing useful insights for the definition of optimized and tailored rehabilitation programs.

Many studies support the hypothesis that the CNS solves the problem of coordinating the activation of several muscles to produce the multi-joint movements assembling a functional task, through the implementation of the so-called Muscle Synergies^19–21^. The latter are extracted from EMG signals and can represent the mechanism used by the cortical sensorimotor areas, brainstem and spinal cord to control groups of muscles concurrently activated to perform a motor task. Each synergy is constituted by two components: the muscle weightings and its temporal activation profiles, the location of which is assumed to be at different levels of the CNS, respectively, in the spinal cord and in cortical/subcortical sensorimotor structures. These units are functional structures related to specific motor patterns, defined as coordinated patterns of muscle activity that are combined flexibly to produce functional motor behaviors^20,22^. Muscle synergies approach has been already used on post-stroke patients both as a metric for motor assessment and to evaluate the effects of rehabilitation^15,23^, revealing that in sub-acute stroke survivors the altered muscle synergies of the paretic arm can be reassembled into those of healthy people following planar robot therapy^15^.

However, the potential changes in muscle synergies induced by robot-assisted therapy in stroke survivors and their difference with respect to those induced by usual care treatment have been poorly investigated and still remain unclear.

Considering all the above mentioned issues, this study reports the results of a prospective, randomized and single-blinded trial. The aim was to evaluate the changes in the motor control mechanisms of post-stroke subjects induced by robot-assisted planar training, with respect to those derived from usual care, during the execution of two non-trained motor tasks typically involved during activity of daily living (i.e. object placing onto a shelf and forearm pronation). We hypothesized that robot-assisted training might provide patients with a better restoring of neurophysiological patterns in terms of upper-limb muscle synergies than conventional therapy due to the strengthening of the specific brain plasticity and connectivity functions related to motor planning and execution^13^.

## Results

### Participant characteristics

**Figure 1:**
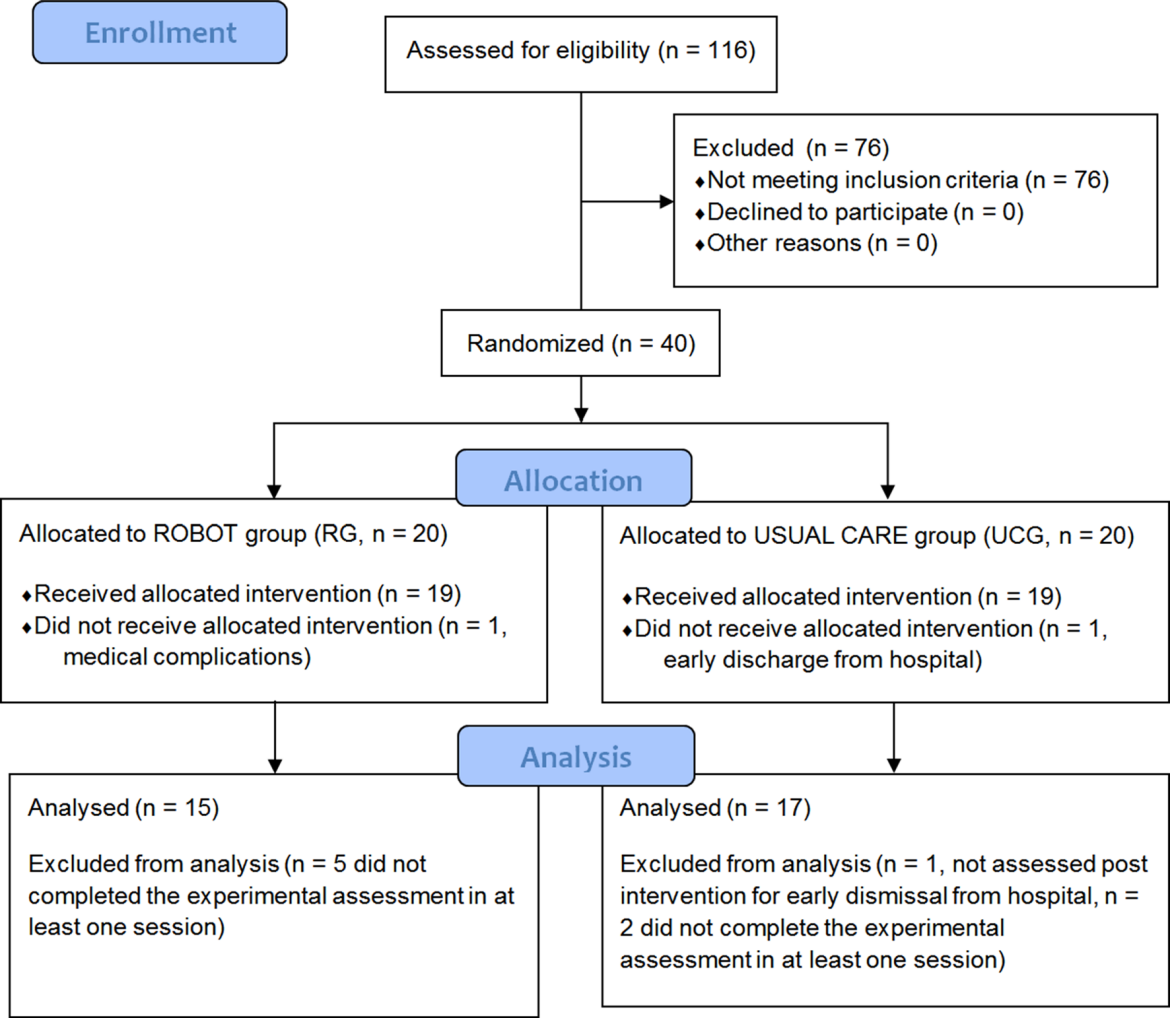
Flow chart of the study.

The baseline demographic and clinical characteristics of the participants in the robot-assisted (RG) and usual care (UCG) groups did not differ significantly (see Table 1). The recruited sample consisted of 32 persons in both chronic (> 3 months post stroke, mean (95%CI) [months], RG 22.4 (5.5–39.3), UCG 65.8 (24.5–107.2), *P* = 0.46) and sub-acute (< = 3 months post stroke, RG 1.4 (0.7–2.2), UCG 1.8 (1.1–2.5), *P* = 0.31) stage of post-stroke^24,25^ recovery (Fig. 1).

**Table 1:** Demographic and clinical features of study participants.

Variable	RG (N = 15)	UCG (N = 17)	*P*-value
	Median (1st–3rd)	Median (1st–3rd)	
Age (year)	68.0 (54.5–74.5)	59.0 (46.9–68.4)	0.22
Time since stroke (months)	7.76 (0.7–27.3)	5.8 (2.9–76.0)	0.59
FM-UE	45 (27.5–49.5)	21 (16.1–41.5)	0.13
	**Number**	**Number**	
**Sex**			0.35
Male	6	9	
Female	9	8	
**Paretic** **arm**			0.38
Right	7	6	
Left	8	11	
**Stroke** **type**			0.44
Hemorrhagic	4	6	
Ischemic	11	11	
**Chronicity** **(**> **3** **months)**			0.61
Chronic	9	10	
Sub-acute	6	7	

FM-UE: Fugl-Meyer Motor Assessment for the Upper Extremities.

*P*-values indicate the results of Mann–Whitney U Test for age and time since stroke, of unpaired t-test for FM-UE and of the Fisher exact test for all the other variables.

All enrolled patients had a contralateral hemiparesis because of an ischemic or hemorrhagic stroke. Regarding the upper limb impairment, this was quite heterogeneous ranging from moderate to severe with a median (first to third quartile) FM-UE score of 32.5 (14.4–49.5).

Lesions were located in the brainstem (N = 2 RG, N = 4 UCG), frontal lobe (9 RG, 10 UCG), parietal lobe (7 RG, 11 UCG), temporal lobe (0 RG, 2 UCG), occipital lobe (0 RG, 1 UCG), internal capsule (2 RG, 1 UCG), thalamus (1 RG, 1 UCG) and basal ganglia (2 RG, 0 UCG).

Nineteen patients (8 RG and 11 UCG) had lesions in two or more regions, with the infarction of the frontoparietal regions being the most common (7 RG, 9 UCG). To note, none of them had bilateral lesions and none of the patients who suffered a brainstem stroke had a bilateral hemiparesis. It is also noteworthy that, no difference in lesion location within the white matter emerged between the treatment groups (chi square test *P* = 0.36).

No adverse events were reported.

### Clinical test FM-UE

Patients underwent the clinical evaluation one day after the end of the intervention.

Following rehabilitation, patients showed an improvement of the motor ability according to the FM-UE scores, regardless of treatment (F(1,29) = 0.34, *P* = 0.56), with comparable change scores in the two groups (Mean(95%CI), RG 7.1 (3.3, 10.9), UCG 5.5 (2.0, 9.1)). In the post training evaluation, all subacute patients, with the exception of one subject in the usual care group, obtained FM-UE values higher than the score estimated on the basis of spontaneous recovery according to the estimates of Duncan et al.^26,27^.

### Instrumented test

All the enrolled post-stroke participants were able to perform autonomously the motor tasks included in the instrumented test both at baseline (T0) and after rehabilitation (T1).

### Baseline

The baseline values of the instrumented outcome measures are reported in Table 2.

**Table 2:** Mean and 95% confidence interval of instrumented parameters for post-stroke subjects at baseline (allocated to Robot Group (RG) and Usual Care Group (UCG)) and for Healthy Subjects (HS).

Outcome measure	RG mean (95% CI)	UCG mean (95% CI)	HS mean (95% CI)
**Object placing task**			
**Amount of elbow extension**^c^ **(°)**			
CA	− 15.6 (− 34.0, 2.7)^a^	− 16.0 (− 33.5, 1.5)^a^	− 58.3 (− 66.7, − 49.9)
IA	− 55.6 (− 66.3, − 45.0)	− 53.9 (− 63.3, − 44.4)	
**Mean RMS of Trunk angle**^**c**^ **Sagittal plane (°)**			
CA	10.9 (7.5,14.3)	10.2 (7.4,13.0)	-
IA	4.6 (2.6,6.5)	4.7 (3.2,6.2)	
**Mean RMS of shoulder angle**^**c**^ **Sagittal plane (°)**			
CA	5.5 (3.3,7.7)	2.9 (2.2,3.6)^b^	*-*
IA	4.8 (2.4,7.2)	5.8 (2.7,8.9)	
**Movement smoothness**^**c**^			
CA	9.5 (7.5,11.6)^a^	10.1 (5.9,14.2)^a^	3.9 (3.2,4.6)
IA	6.3 (4.3,8.2)	7.8 (6.0,9.6)^b^	
**Pronation task**			
**Amount of Wrist Pronation**^d^** (°)**			
CA	24.6 (15.4,33.7)^a^	15.3 (7.0,23.5)^a^	54.9 (37.2,72.6)
IA	44.6 (32.7,56.6)	43.3 (33.9,52.7)^a^	
**Mean RMS of Trunk Angle**^**c**^ **Horizontal Plane (°)**			
CA	4.7 (2.0,7.4)	4.5 (2.6,6.5)	–
IA	3.2 (0.8,7.3)	1.8 (1.3,2.3)	
**Mean RMS shoulder angle**^**c**^ **Frontal Plane (°)**			
CA	6.8 (3.7,10.0)	5.5 (3.3,7.7)	**–**
IA	9.6 (2.3,16.9)	5.2 (3.5,6.9)	
**Movement smoothness**^**c**^			
CA	6.6 (5.0,8.2)^a^	5.2 (3.8,6.7)^b^	3.0 (2.3,3.8)
IA	4.4 (3.1,5.7)	6.7 (4.5,8.8)^a^	

^a^Statistically significant different with respect to HS.

^b^Statistically significant different with respect to RG.

^c^Lower values indicate better performance.

^d^Higher values indicate better performance.

RMS, root mean square; CA, Contralesional arm; IA, Ipsilesional arm.

#### Object placing task, post-stroke patients versus healthy subjects

At baseline the contralesional arm (i.e. plegic arm) of patients with stroke (RG and UCG) had statistically significant different values of the Amount of Elbow Extension (Table 2) and Movement Smoothness with respect to those of HS. The mean RMS of the trunk and shoulder joints showed values greater than zero indicating a deviation from the normative reference.

No difference emerged for the parameters of Elbow Extension and of Movement Smoothness related to the ipsilesional arm compared to HS. Instead the mean RMS of trunk and shoulder joints of this arm had values greater than zero.

#### Forearm pronation task, post-stroke patients versus healthy subjects

At baseline the contralesional arm of patients with stroke (RG and UCG) showed statistically significant different value of the Amount of Wrist Pronation (Table 2) with respect to that of HS. Only post-stroke patients belonging to RG group showed abnormal values of Movement Smoothness with respect to HS. The mean RMS of the trunk and shoulder joints had values greater than zero indicating a deviation from the normative reference.

Regarding the ipsilesional arm only post-stroke patients belonging to the UCG group showed different values of the Amount of Wrist Pronation (Table 3) and of Movement Smoothness with respect to those of HS. Also the mean RMS of the trunk and shoulder joints of this arm had values greater than zero.

**Table 3:** Mean and 95% confidence interval (CI) of change scores (post-baseline values) of kinematic parameters for Robot group (RG) and Usual care group (UCG).

Outcome Measure	RG Mean (95% CI)	UCG Mean (95% CI)	P Value Between-group difference
**Object placing task**			
**Amount of Elbow Extension** **(°)**^a^			
CA	− 27.8 (− 41.6,14.0)	− 7.7 (− 20.6,5.3)	*P* = 0.037
IA	− 0.7 (− 9.7, 8.2)	3.6 (− 4.8, 12.0)	*P* = 0.480
**Mean RMS of trunk angle sagittal plane (°)**^**a**^			
CA	− 3.5 (− 5.7, − 1.2)	0.4 (− 1.7, 2.5)	*P* = 0.018
IA	0.3 (− 1.1, 1.8)	1.0 (*-*0.3, 2.4)	*P* = 0.485
**Mean RMS of shoulder angle sagittal plane (°)**^**a**^			
CA	1.8 (0.0, 3.5)	0.0 (− 1.7, 1.6)	*P* = 0.150
IA	0.07 (*-*1.52, 1.65)	− 0.08 (− 1.57,1.41)	*P* = 0.894
**Movement smoothness**^**a**^			
CA	− 1.4 (− 3.6,0.9)	− 1.8 (− 3.9, 0.3)	*P* = 0.768
IA	− 1.1 (− 2.5, 0.3)	− 0.8 (− 2.1, 0.5)	*P* = 0.722
**Forearm pronation task**			
**Amount of forearm pronation (°)**^b^			
CA	24.3 (14.4, 34.3)	9.4 (0.1, 18.7)	*P* = 0.036
IA	4.7 (− 3.4, 12.9)	− 2.4 (-− 10.1, 5.3)	*P* = 0.205
**Mean RMS of trunk angle horizontal plane (°)**^**a**^			
CA	− 0.3 (− 1.7, 1.2)	− 0.5 (− 1.9, 0.9)	*P* = 0.802
IA	− 0.8 (− 1.5, − 0.1)	− 0.2 (− 0.9, 0.4)	*P* = 0.209
**Mean RMS shoulder angle frontal plane (°)**^**a**^			
CA	1.3 (− 0.1, 2.7)	− 0.8 (− 2.1, 0.5)	*P* = 0.034
IA	− 0.9 (*-*3.1, 1.2)	*-*1.6 (*-*3.6, 0.4)	*P* = 0.665
**Movement smoothness**^**a**^			
CA	2.8 (0.6, 5.1)	0.0 (− 2.1, 2.1)	*P* = 0.069
IA	− 1.1 (− 2.5, 0.2)	− 1.4 (− 2.6, − 0.1)	*P* = 0.801

RMS, root mean square; CA, Contralesional arm; IA, Ipsilesional arm.

*P*-values indicate the results of the comparison between RG and UCG according to the analysis of covariance (ANCOVA), adjusted for baseline score.

^a^Lower scores indicate better performance.

^b^Higher scores indicate better performance.

#### Object placing and forearm pronation task, post-stroke patients RG versus UCG

Within the framework of comparisons between treatment groups, the instrumented outcome measures related to the RMS values were comparable for both arms in both tasks, with the exception of the RMS of shoulder joint of the contralateral arm during the object placing task. For this parameter and the Movement Smoothness of the pronation task, the UCG patients showed lower values than RG patients indicating a smaller deviation from the normative data.

### T0 versus T1

Table 3 reports the pre to post change scores of the calculated kinematic parameters.

#### Object placing task

For what concerns the performance of the contralesional side during the object placing task, the change score of the Amount of Elbow Extension (Table 3) was statistically significantly different between groups, in favor of the robot treatment (F(1,29) = 4.76, *P* = 0.037), with the RG group showing a larger elbow extension after treatment with respect to UCG group. The RG attained a larger improvement also in the trunk movement during the performance (Table 3, Mean RMS of Trunk Angle, F(1,29) = 6.30, *P* = 0.018), as demonstrated by the reduction of the deviation from the normative values (Table 3). No difference between groups emerged regarding the pre to post change of the deviation of the angular curve (RMS value) of shoulder from normative values.

The pre to post change score of all movement smoothness parameters showed negative values indicating an improvement of the movement execution, with no significant difference between groups.

As regard the ipsilesional arm, no difference between groups emerged in the change score of any kinematic parameter.

#### Forearm pronation task

For what concerns the performance of the contralesional arm, the change score of the Amount of Wrist Pronation (Table 3) was statistically significantly different between groups in favor of the robot treatment (F(1,29) = 4.81, *P* = 0.036). Conversely, the change score of the Mean RMS of Shoulder Angle (Table 3) was significantly different between groups in favor of the UCG, that was the only group who showed a pre to post decrease of the deviation from the normative curves.

As regards the smoothness of the movement, the change score of the RG was significantly worse than the UCG group, since the former had markedly worsened, while the latter did not change from pre to post.

Regarding the ipsilesional arm no difference between groups emerged in the kinematic parameters.

### Muscle synergies in healthy and post stroke subjects

#### Extraction of muscle synergies

In this study we adopted the 90% R^2^ criterion in the extraction of task-specific synergies in both healthy and stroke subjects.

The number of extracted synergies was not significantly different between healthy and post-stroke subjects, considering for the latter both the pre- and the post-treatment evaluation. Moreover, in post-stroke subjects no changes emerged in the paired analysis comparing the synergies extracted from pre and post evaluations, both on the ipsi and the contralateral arm. For this reason, two synergies, corresponding to the rounded average across groups and repeated assessments, was retained for all subjects for both arms.

The organization of the two extracted muscle synergies is described in the following paragraphs for each task.

1. Object placing task
  Synergies 1 involved the UPTR, RHMA, PEMI, INFR, BRRA, SUPI and PRON muscles. This axial-to-proximal synergy was active during the entire task, mostly in the first half of the execution of the movement. This synergy facilitated the stabilization of the trunk and forearm (Fig. 2, C ID1).
  Synergies 2 involved the TBLH, TBMH, BBSH, BBLH, ANDE, PODE, LADE and BRAC muscles. This axial-to-proximal synergy was mainly active during the final part of the task and was responsible of the functional execution of the movement. This synergy controlled mainly the extension and flexion, respectively, of the elbow and of the shoulder (Fig. 2, C ID2).

**Figure 2:**
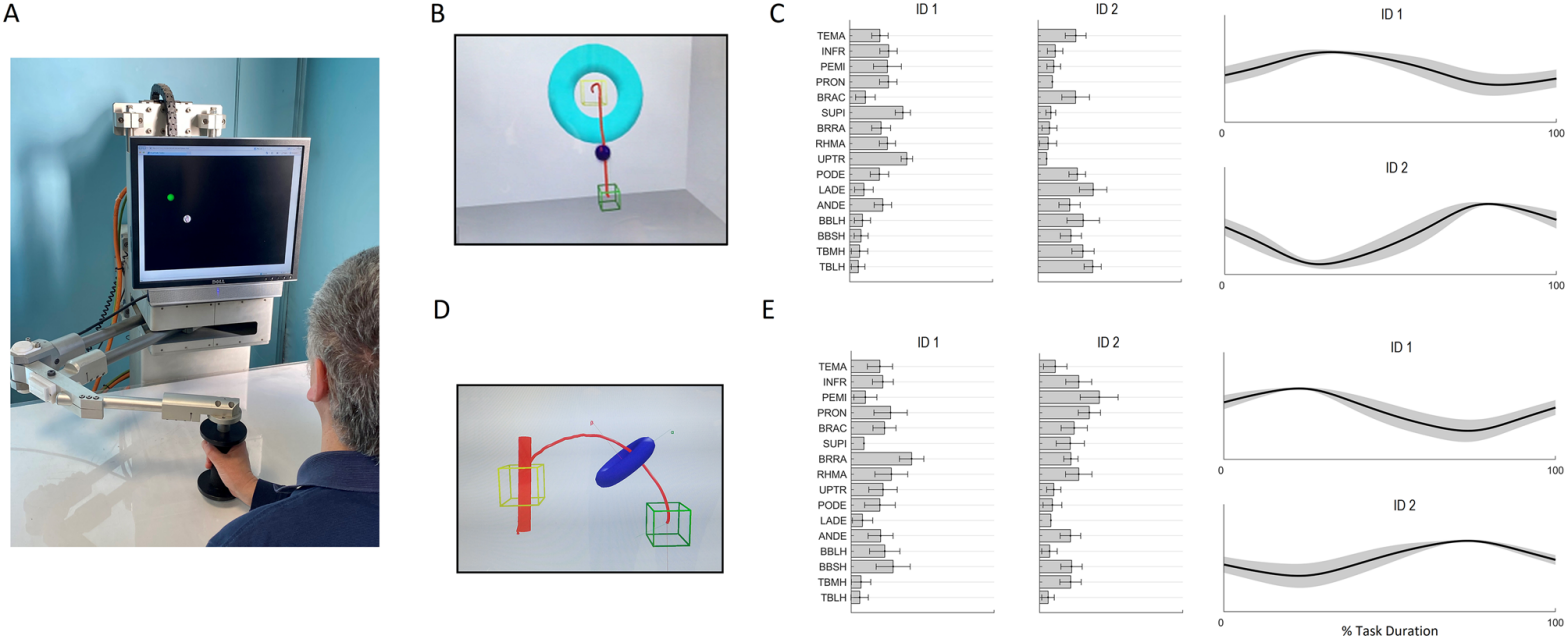
(**A**) In the left panel a subject using the robot device is reported. The central panel reports the virtual scenario shown to the subject during the object placing (**B**) and forearm pronation (**D**) tasks. The blue ball/donuts represents hand’s movement, the green box is the starting position, the yellow box is the target position. The red line shows the trajectory of one representative subject (not shown during the test). The right panel reports the muscle synergies extracted on healthy subjects for object placing (**C**) and forearm pronation (**E**) tasks. The bars indicate the group mean and ± 1 SD of motor weightings, while the solid line shows the group average activation profile with the grey area representing ± 1 SD of profiles inter-subject variability.
2. Forearm pronation task
  Synergies 1 involved TEMA, PRON, BRRA, UPTR, PODE, BBSH and BBLH muscles. This proximal-to-distal synergy was active during the entire task in phase opposition with the other synergies. This synergy facilitated the stabilization of the trunk and arm (Fig. 2, E ID1).
  Synergies 2 involved PEMI, PRON, SUPI, BRRA, BBSH and TBMH muscles. This proximal-to-distal synergy was active during the entire task in phase opposition with the other synergies. This synergy control mainly the supination-to-pronation of the forearm and the adduction/abduction rotation of arm with respect to trunk (Fig. 2, E ID2).

### Organization and temporal activation of the muscle synergies (activation profile, C and weightings, W)

The aim of the work was to investigate muscle synergies changes related to the type of therapy, robotic or usual care rehabilitation.

The indices of similarity between muscle synergies weightings and activation profile of post-stroke patients and those of healthy subjects were normally distributed and their variances were homogeneous. Therefore, we ran an ANOVA test to characterize the differences between the treatment groups at baseline and an ANCOVA test with “Therapy” as the between-group factor and the baseline assessment as covariate.

#### Comparison at baseline

At baseline the similarity of weightings (W) in post-stroke persons was mostly preserved for both arms in both tasks (Table 4, Pre treatment values of Sim W about 0.70), while the activation profiles were altered (Sim C largely < 0.70), with the exception of the activation profiles of the ipsilesional side during the object placing task (Sim C > 0.89). The comparison of similarity of motor weightings and activation profiles between treatment groups (UCG vs RG) showed not significantly different values (*P* > 0.05) for both arms and tasks, with the exception of motor weightings of synergy 1 (W1) in the ipsilesional arm for the object placing task, that showed a larger similarity in the UCG with respect to RG (F(1,30) = 6.16, *P* = 0.019).

**Table 4:** Mean and 95% confidence interval (CI) of pre and post values of the similarity of muscle synergies for Robot group (RG) and Usual care group (UCG).

Outcome measures	RG Mean (95%CI)		UCG Mean (95%CI)	
	Pre	Post	Pre	Post
**Object placing task**				
Sim W1				
CA	0.69 (0.66,0.73)	0.72 (0.69,0.75)	0.69 (0.65,0.72)	0.70 (0.67,0.73)
IA	0.71 (0.68,0.73)	0.73 (0.71,0.76)	0.75 (0.72,0.77)^a^	0.71 (0.68,0.74)
Sim W2				
CA	0.71 (0.68,0.74)	0.73 (0.70,0.77)	0.72 (0.70,0.75)	0.68 (0.65,0.71)
IA	0.76 (0.74,0.79)	0.77 (0.75,0.80)	0.76 (0.74,0.79)	0.76 (0.74,0.79)
Sim C1				
CA	0.19 (− 0.23,0.62)	0.63 (0.27,1.00)	0.19 (− 0.21,0.59)	0.29 (− 0.05,0.64)
IA	0.92 (0.86,0.98)	0.90 (0.81,0.98)	0.89 (0.83,0.94)	0.84 (0.76,0.92)
Sim C2				
CA	0.21 (− 0.22,0.65)	0.78 (0.43,1.12)	0.17 (− 0.24,0.58)	0.37 (0.04,0.69)
IA	0.96 (0.92,0.99)	0.94 (0.88,0.99)	0.94 (0.91,0.97)	0.91 (0.85,0.96)
**Pronation task**				
Sim W1				
CA	0.69 (0.64,0.73)	0.70 (0.66,0.73)	0.68 (0.64,0.73)	0.69 (0.66,0.72
IA	0.69 (0.65,0.73)	0.70 (0.67,0.73)	0.68 (0.64,0.72)	0.69 (0.66,0.72
Sim W2				
CA	0.66 (0.62,0.70)	0.67 (0.64,0.71)	0.66 (0.63,0.70)	0.69 (0.65,0.72)
IA	0.65 (0.61,0.69)	0.71 (0.68,0.74)	0.67 (0.63,0.71)	0.68 (0.65,0.70)
Sim C1				
CA	0.48 (0.09,0.86)	0.20 (− 0.27,0.68)	0.53 (0.17,0.89)	0.17 (− 0.28,0.61)
IA	0.70 (0.40,1.01)	0.45 (0.06,0.84)	0.79 (0.50,1.08)	0.58 (0.22,0.95)
Sim C2				
CA	0.55 (0.18,0.92)	0.25 (− 0.21,0.72)	0.63 (0.28,0.98)	0.23 (− 0.21,0.66)
IA	0.59 (0.22,0.95)	0.44 (0.05,0.83)	0.76 (0.46,1.07)	0.68 (0.36,1.00)

Sim W1: similarity of weightings of muscle synergy 1 with respect to those of healthy subjects.

Sim C1: similarity of activation profile of muscle synergy 1with respect to those of healthy subjects.

Sim W2: similarity of weightings of muscle synergy 2 with respect to those of healthy subjects.

Sim C2: similarity of activation profile of muscle synergy 2 with respect to those of healthy subjects.

CA, Contralesional Arm; IA, Ipsilesional Arm.

^a^Statistically significant differences with respect to RG at baseline.

#### Effect of treatments

**Figure 3:**
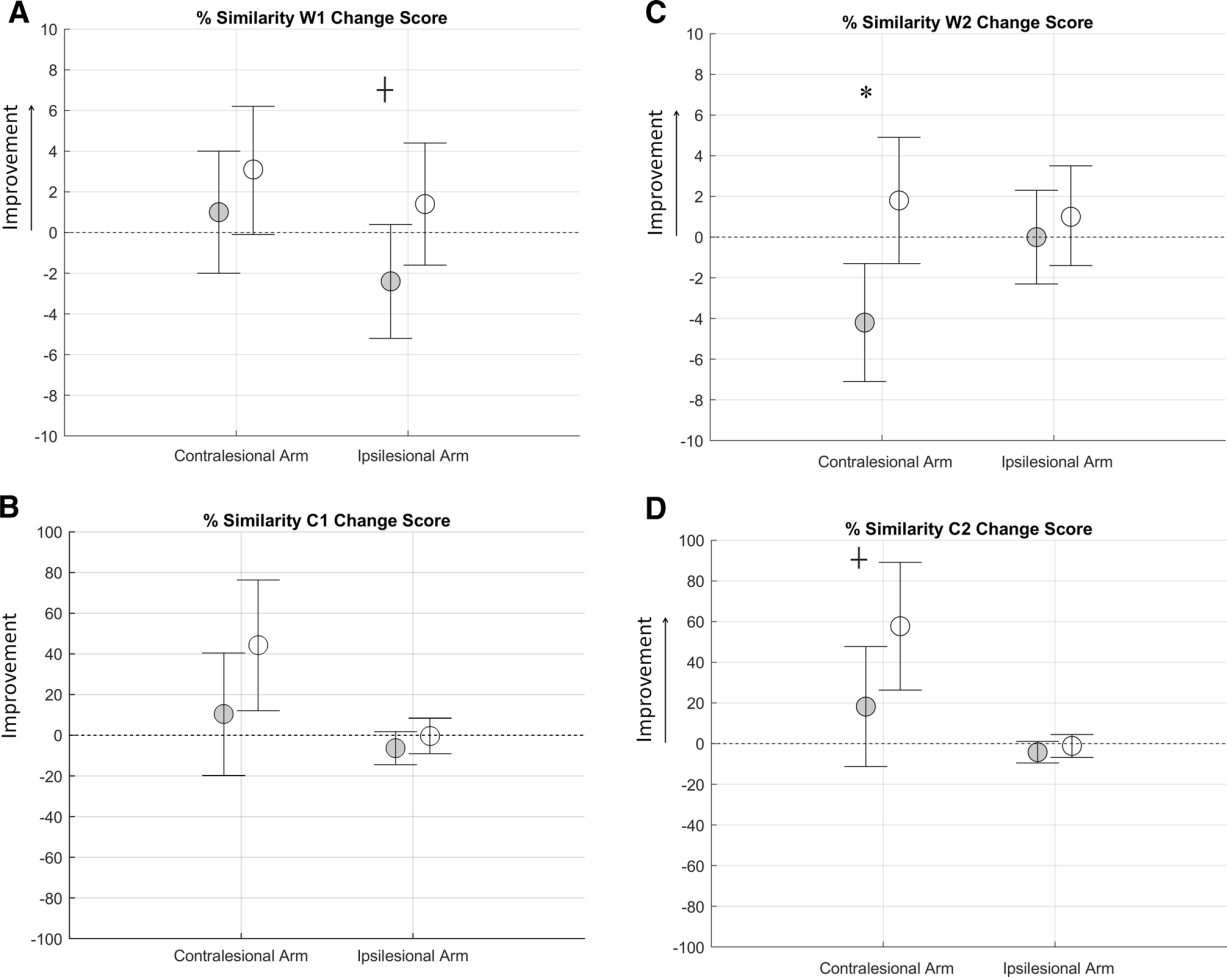
Pre to post change scores from baseline attained by post-stroke participants after robot therapy (RG, white circles) and usual care intervention (UCG, gray circles) during the object placing task for muscle weightings (top panels) and activation profiles (bottom panels) of synergy 1 (**B**–**D**) and 2 (**C**–**E**). Circles and whiskers represent, respectively, mean change score and 95% confidence interval adjusted for baseline score through ANCOVA procedure. *P < 0.05 (RG vs. UCG, ANCOVA test). + 0.05 ≤ *P* < 0.1 (RG vs UCG, ANCOVA test).

In the object placing task, ANCOVA did not demonstrate a significant difference on the effects of the two interventions in terms of similarity of muscle synergy 1 for both the muscle weightings (Fig. 3A) and the activation profile (Fig. 3B) for both arms, with the exception of the similarity of muscle weightings of ipsilesional arm in favor of the RG group (F(1,29) = 3.38, *P* = 0.07). For what concerns the muscle synergy 2, there was a significantly greater effect in both muscle weighting (Fig. 3C F(1,29) = 8.33, *P* < 0.01) and activation profile ( Fig. 3D F(1,29) = 3.52, *P* = 0.07) in the RG than in the UCG group, for the contralesional arm only.

**Figure 4:**
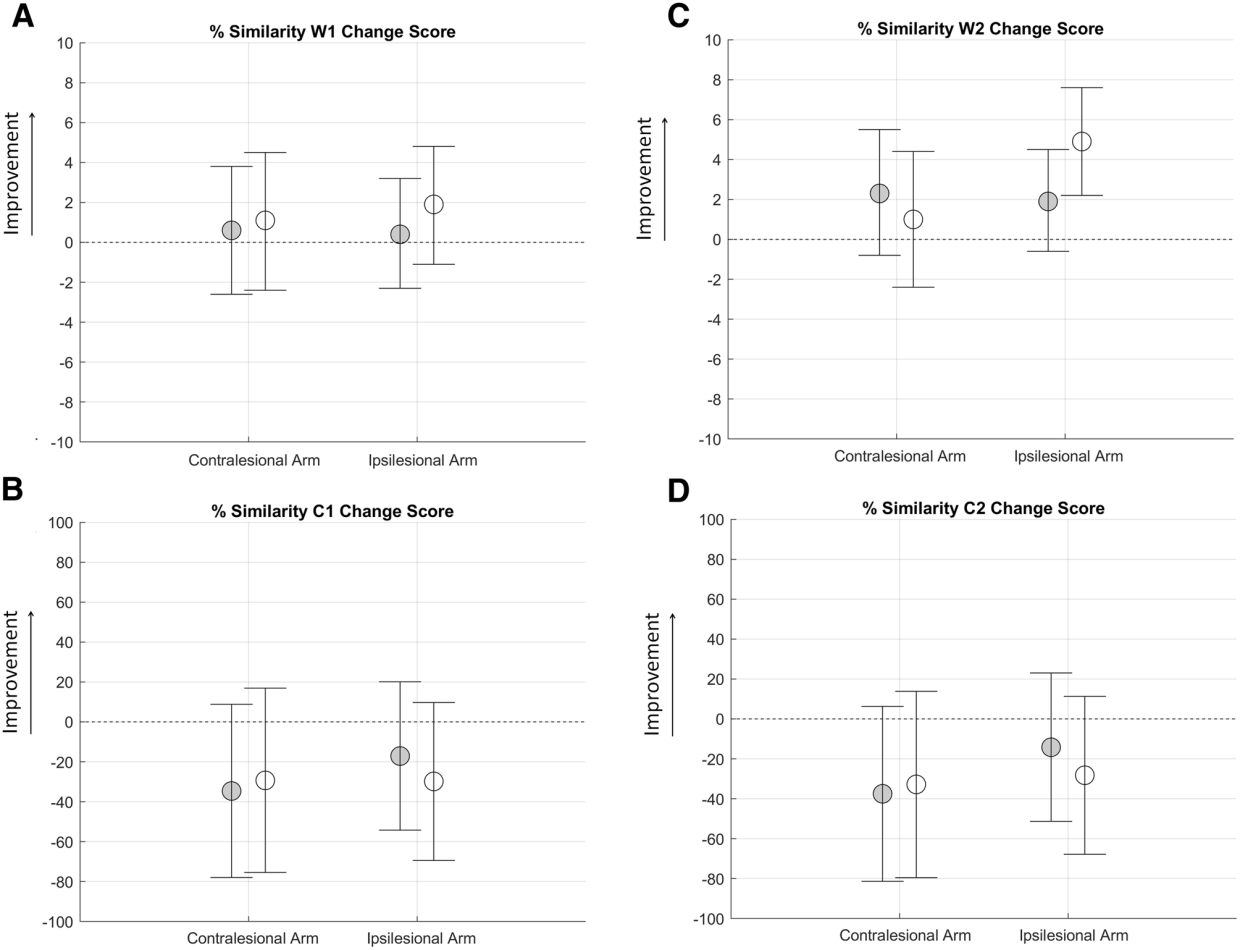
Pre to post change scores from baseline attained by post-stroke participants after robot therapy (RG, white circles) and usual care intervention (UCG, gray circles) during the forearm pronation task for muscle weightings (top panels) and activation profiles (bottom panels) of synergy 1 (**B**–**D**) and 2 (**C**–**E**). Circles and whiskers represent, respectively, mean change score and 95% confidence interval adjusted for baseline score through analysis of covariance ANCOVA test. *P < 0.05 (RG vs. UCG, ANCOVA test). + 0.05 ≤ *P* < 0.1 (RG vs UCG, ANCOVA test).

In the forearm pronation task, the analysis showed a comparable positive effect of the two interventions in the weightings (W) of both muscle synergies for both arms (Fig. 4A,C). Conversely, a negative effect of both interventions emerged on the activation profile of both muscle synergies for both arms (Fig. 4B,D).

## Discussion

At the baseline, post-stroke subjects performed the two considered motor tasks less effectively than healthy subjects (Table 2): they showed a reduction of elbow extension during the object placing task and a reduction of wrist pronation during the pronation task. Moreover, in both tasks they moved less smoothly than healthy controls and with an abnormal profile of trunk and shoulder movements. Looking at the motor control (i.e. muscle synergies) behind their kinematic performance, the number of synergies were comparable with those of healthy subjects. The structures of synergies, in terms of muscle weightings (W), were mostly preserved for both the contralesional and the ipsilesional side, while the activation profiles were mostly altered.

Some studies in the framework of muscle synergies, have explained abnormal motor performance by an alteration in the structure of modules (merging/fractionalization of the physiological modules found in healthy subjects), abnormal activation profile of normal modules, or both. However, a large number of the published studies on post-stroke subjects with disease severity comparable to the population here presented have supported the hypothesis of preservation of a low-dimensional modular organization of muscle synergies. In these studies, the abnormal motor performance was mostly attributed to the abnormal alteration of activations profiles (recruitment) of motor modules. One of the first studies on upper limb synergies had identified three distinct patterns of motor coordination, reflecting preservation of normal muscle synergies in less impaired individuals and merging or fractionation of normal muscle synergies in subjects with more severe deficits^28^. Instead our findings related to the number of synergies and similarities of the modules do not seem to suggest relevant modifications in the motor modules, as already suggested^29^. This preservation in motor control is in line with the hypothesis that the motor modules are fixed structures embedded within the spinal circuit^20,30,31^, and the stroke involves important alterations at the cortical/subcortical level but not at the spinal one.

The preservation of module organization occurred despite the here considered subjects having lesions over different cortical/subcortical locations, further confirming the hypothesis that muscle synergies are structured most likely by neuronal networks downstream of the cortex, such as the spinal interneuronal circuitries. The module conservation is more evident for synergies that control the dynamic movement involving axial and proximal muscles related to the elbow and shoulder joints (Table 3, object placing task, W2 CA 0.71 IA 0.76). While slight structural changes emerged in synergies that were involved in the required rotation of the arm and forearm for medial and upward movement, regardless of the arm, ipsilesional or contralesional side (Table 3, forearm pronation, W2 CA 0.66; IA 0.66). The more preserved module for axial-to-proximal muscles control (observed in the object placing task) rather than for proximal-to-distal muscles (observed in the pronation task) might reflect the specific functional anatomy of the different components of the corticospinal tract (CST). Differently from lateral CST, which represents the major component of the CST devoted to the control of contralateral distal muscles of the arm, the anterior CST controls mainly axial and proximal muscles via bilateral projections at spinal level^32^. In this view, the anterior CST contralateral to the lesioned hemisphere can recruit the ipsilateral proximal and axial muscles of the contralesional side resembling for this aspect a pre-stroke condition.

Our study highlighted that the abnormal coupling of upper limb joints that post-stroke participants exhibited for their contralesional arms was mainly the result of alterations of the control signals of CNS. In fact, in line with the anatomo-functional organization of corticospinal tract, which is mainly devoted to the axial-to-proximal control of contralateral upper-limb^33^, the synergies activation profiles of the contralesional arm were abnormal, especially in the object placing task (Table 3, sim C1 and C2 < 0.21). On the other hand, the activation profiles of the ipsilesional arm in the same task were very similar to the normative profiles found on healthy subjects (Table 3, sim C1 and C2 > 0.89).

Lower values of similarity of the activation profiles were shown in the execution of the forearm pronation task, especially for the ipsilesional arm (Table 3, sim C1 and C2 0.50 and 0.59). This finding supports the evidence of other studies, which highlighted abnormalities in the ipsilesional arm after stroke, especially for the control of forearm distal movements^34–37^. A possible explanation is that stroke affects not only the lateral CST devoted to ipsilateral distal control but also the sensorimotor circuits altering the activity of the contralateral sensorimotor areas through transcallosal inhibitory connections^38,39^. The interhemispheric inhibitory interactions have been directly studied in post-stroke patients showing that these subjects abnormally increased transcallosal inhibition from the healthy hemisphere onto the injured side relative to healthy subjects^40,41^. This was also recently demonstrated by Spalletti et al. by investigating the motor recovery in rodent model inducing focal ischemic lesion in the primary motor cortex of mices^42^. This study further confirmed that the healthy contralesional hemisphere exerts an increased transcallosal inhibition over the spared perilesional tissue. The normalization of the interhemispheric imbalance through transcranial magnetic stimulation protocol could improve the motor function of these subjects^41–44^.

Involvement of the ipsilesional arm has several implications for data analysis and rehabilitation planning, (1) only healthy subjects should be used as control group in the investigation of muscle synergies after stroke and not the patient’s ipsilesional arm and (2) the bilateral hemispheric control of the distal upper limb should be taken into consideration when planning rehabilitation programs.

The improvement of upper limb movement following rehabilitation is often used as the key to evaluate the level of success of the treatment. Motor skill reacquisition is defined as improvement on an outcome measure either at the level of functions or at the level of activities. Improvement after stroke can be linked to: (1) true (neurological) recovery reflecting the return or restitution (or repair) of body functions (or reduction of impairments), which results in the reappearance of the as physiological as possible task performance^45^ and (2) skill reacquisition through motor compensation at an activity level, which can be defined as the appearance of a different motor patterns resulting from compensation by the remaining working motor elements at the level of body function, using different articular joint or body segments to perform the task^46^.

In post-stroke patients, the values at baseline of RMS-based parameters (not close to 0) and of those related to the pronation and elbow extension (Table 4) indicate that their body segments performed the tasks in a different way compared to the physiological patterns.

In particular the baseline assessment showed that the post-stroke subjects executed the tasks with a significant impairment of the amount of elbow extension (object placing task) and pronation (forearm pronation task), as found in previous studies^5,47^. These different motor patterns compared to those of healthy subjects seem to be attributable to compensation schemes, as highlighted by the alterations of the activation profiles of the synergies, which reflect the activity of the brain regions that modulate the movement (Table 2).

Within the framework of proximal upper limb movement (object placing), a global evaluation of the movement, such as the smoothness calculated with reference to the end effector (hand), indicates that both treatments have a comparable positive effect on movement. Looking into the details, for what concerns the elbow extension, the RG attained a significantly larger improvement with respect to UCG (Table 3, Amount of elbow extension) with a better trunk angular profile, often used to compensate the movement impairment^11^. The most important finding of this study is that this kinematic improvements, favoring the RG, were associated to an improvement of the activation profile of synergy 2, that controls the dynamic execution of the task (Table 4, Sim C2, Fig. 3D). The more physiological activation pattern of RG leads to a better structural activation of the motor module associated with it (Table 4, Sim W2, Fig. 3C). This did not occur in the UCG group where, despite a slight improvement in the activation profile, there was a worsening of the similarity of the motor module, suggesting probably that different motor representation patterns may have been activated.

The improvement of the elbow kinematic and of the synergy 2, in favor of the RG, suggests that robot-assisted rehabilitation supports true neurological recovery, especially for the upper limb proximal district (shoulder/elbow). This was also supported by the recent evidences from the rodent model of stroke where it seems that, following rehabilitation, temporal features of cortical activation recover toward pre-stroke conditions through the progressive formation of a new motor representation near the injured area^48^.

Within the framework of arm distal movement (forearm pronation), the contralesional arm change score of movement smoothness favors the UCG because of a worsening in the RG. This condition also occurs for the RMS parameters related to shoulder joint. In contrast to the sentence above, the RG attained significantly a larger improvement with respect to UCG in terms of pronation movement. Since both treatment groups had a worsening in the activation profile of both synergies (Fig. 4B,C), the improvement could be the result of a positive effect of the achieved proximal improvement (i.e. elbow extension). In fact, post-stroke subjects often activate abnormal connection between elbow and forearm through a stereotypical extensor synergy (characterized by simultaneous shoulder adduction, elbow extension, and forearm pronation)^49^. After all, in most of the cases forearm rehabilitation in post-stroke subjects does not bring to a full recovery and the reasons for explaining this non-recovery are still unknown. It has recently been hypothesized that hyperactivity of the contralesional hemisphere after stroke could be a cause. In fact on rodent models, robotic therapy combined with the pharmacological inhibition of the contralesional primary motor cortex produced a recovery of the forearm motor function restoring pre-stroke cortical activations^48^. This should be further investigated in human beings.

Recent reviews of robot treatment have shown non-significant improvements or small effects on daily function after upper limb robotic rehabilitation in patients with stroke^8,9,50^. Major goals of stroke rehabilitation are to improve not only motor function but also functional performance on daily activities. The study here presented provided the important finding that the robot treatment improves the motor control and motor function succeeding in the transfer of achieved recovery to an activity of daily living (object placing and forearm pronation that simulate the transport of an object onto a shelf and the turning of the pages of a newspaper, respectively).

In conclusion, the present findings highlighted that muscle synergies assessment can detect the reorganization of upper limb muscle coordination during motor recovery after stroke, something that cannot be captured by the clinical scales.

Training provided by a planar robot can successfully modify abnormal muscle synergies to resemble the healthy ones in the proximal district (i.e. trunk, shoulder and elbow joints). However, there have been negative effects in the control of the distal district (i.e. forearm, wrist joint) which could be improved by adding other forearm-specific rehabilitation (e.g. electrostimulation, transcranial magnetic stimulation or transcranial electrical stimulation) or distally targeted robot-assisted therapy (e.g. to the hand or the wrist). Furthermore, the evident alterations in the ipsilesional arm activation patterns, despite the good motor performance, suggest that this aspect must be further investigated and taken into consideration during rehabilitation.

Tracking the changes of abnormal muscle synergies of both arms during motor recovery and rehabilitation may provide new insights into the neural reorganization after stroke and may help to define the nature and the timing of therapeutic interventions and to tailor it to the patient with greater effectiveness.

### Study limitation

First, the size of the examined sample was dimensioned only according to guidelines for randomized controlled trial at the demonstration-of-concept stage and should be increased to detect a difference in the primary clinical outcome measure. A second limitation is the lack of follow-up assessments that did not allow the analysis of retention of training effects. Future studies on a larger sample, including also follow-up assessments, should be performed to corroborate present findings and assess long-term training effects. Third, the extraction of muscle synergies is dependent on methodological aspects. Finally, although 16 out of 32 subjects had a fronto parietal lesion, giving a certain degree of homogeneity to the study, the sample includes subjects also having cortical or subcortical lesions randomly distributed between the treatment groups. This variability of the lesion location could involve different mechanisms of recovery. However, the absence of a significant difference between groups in lesion location and the presence of about 50% of patients with parieto-frontal lesion makes us reasonably conclude that this does not misleadingly affect our results and interpretations. Aware of the above limitations, future studies are required to better investigate these aspects by improving the methodological aspect by grouping different sample of patients based on different lesion localization.

## Methods

### Study design

As detailed in our previous scientific publication^11^, the present study is part of the MOSE study (ClinicalTrial.gov, NCT03530358, 21/05/2018), a multicenter center randomized controlled trial. The IRCCS San Camillo Hospital, Venice, Italy (Center 1) tested the efficacy of virtual reality-based training as an approach for the stroke upper limb rehabilitation, while the IRCCS Don Carlo Gnocchi Foundation, Milan, Italy (Center 2) tested the robot-assisted therapy.

Each center carried out a single-blind two-arm randomized 1:1 controlled trial. Only the Center 2 acquired the kinematics of upper limb simultaneously to the recording of EMG signal during the execution of the functional motor tasks. Thus, in the present study only data from Center 2 are analyzed and presented.

Specifically, we compared the effects of robot-assisted training and usual care on muscle synergies and kinematics of upper limb in all post-stroke subjects recruited and randomized at Center 2, who were able to perform both tasks, focused on movements of object placing and forearm pronation (see the flow chart of the study, Fig. 1).

### Participants

One hundred and sixteen adult post stroke subjects were evaluated for eligibility at Center 2 in the period from March 2015 to November 2017. Among these subjects, 40 post-stroke adults matched the criteria, as detailed in our previous study^11^. From these enrolled participants, 32 subjects who underwent a complete movement analysis of the upper limbs (electromyography and kinematics) pre- and post-intervention were considered for the present study (Fig. 1).

Inclusion criteria were: age > 18 years, first-time ischemic or hemorrhagic stroke, a National Institute of Health Stroke Scale Motor Arm score ranging from 1 to 3 and a score higher than 6 out of 66 points on the Fugl-Meyer Motor Assessment of Upper Extremity (FM-UE) scale. Exclusion criteria were: presence of a moderate cognitive decline defined as a Mini Mental State Examination score < 20 points, evidence of severe verbal comprehension deficit, apraxia and/or visuospatial neglect as assessed through neurological examination, report in the patient’s clinical history or evidence from the neurological examination of behavioral disturbances (i.e. delusions, aggressiveness and severe apathy/depression) that could affect compliance with the rehabilitation programs, presence of non-stabilized fractures, presence of traumatic brain injury, presence of drug resistant epilepsy.

Participants were consecutively randomized to the Robot Group (RG) or the Usual Care Group (UCG) though a simple random number sequence generated by a computer. The procedure of randomization was stratified according to disease onset (≤ 3 months or > 3 months) to ensure that the patients’ chronicity in each group was comparable. To ensure concealed allocation, the investigator responsible for randomization had no clinical role in the study.

A sample of ten healthy subjects (HS), without any musculoskeletal or neurological disorders, provided normative data related to joint kinematics and muscle synergies during the considered functional tasks (see section Muscle Synergies Assessment)^11^.

All participants gave their written informed consent to the study that was conformed to the Declaration of Helsinki and was approved by the ethical committee of IRCCS Don Carlo Gnocchi Foundation, Milan, Italy (session October 15, 2014).

### Intervention

Subjects in both Robot and Usual Care groups received rehabilitation treatments for upper limb, consisting of 20 sessions, each lasting 45 min, 5 times a week, from trained physiotherapists.

The interventions have been previously published^11^ but are presented briefly below. The robot-assisted treatment (BRACCIO DI FERRO, Celin s.r.l., Italy, Fig. 2A), fully described in literature^11^, consisted in controlling the position of the end-effector of a planar robot with the contralesional arm (i.e. paretic limb), while taking it forward and backward from a central position to five targets placed randomly around a circumference with a radius of 20 cm. The robotic system allowed the execution of reaching movements in two force modes, assist-as-needed or resistive, which were chosen by the physiotherapist during each session based on subject’s residual skill/improvement.

Subjects in the UCG underwent usual care arm-specific physiotherapy, that in the current study consisted of passive and active mobilization of scapula, shoulder, elbow and wrist, followed by task-oriented exercises that incorporated single or multi-joint movements aimed at improving arm functionality. Exercises were tailored to patients’ abilities and progression was obtained by increasing range of motion, number of repetitions and muscular coordination requests.

### Outcome measures

#### Motor performance FM-UE assessment

Subjects were clinically evaluated by a trained examiner, unaware of group assignment, at baseline (T0) and post-training (T1). The Fugl-Meyer scale (FM)^51^ uses a 3-point ordinal scale to assess the level of sensorimotor function in the more affected upper extremity (UE). We used only the UE motor function items. The maximum total motor score is 66, with higher scores indicating better motor performances.

#### Instrumental assessment

All participants (RG and UCG) were required to perform two 3D functional motor tasks with both arms (ipsilesional and contralesional) separately. The object placing and forearm pronation tasks were recorded at T0 and T1 to assess the effects of rehabilitation on non-trained functional tasks, typical of activity of daily living, which allow the assessment of the upper limb performance of both proximal (shoulder and elbow) and distal (forearm and wrist) districts. The test was executed using the virtual reality system VRRS (Khymeia Group Ltd., Italy). For both tasks the subject was seated in front of a screen grasping the VRRS electromagnetic sensor with the examined hand. The movement of the sensor (i.e. the hand) was represented by a virtual object on the screen. After the placement of the hand in the starting position (see below), the subject was required to move the virtual object according to the scenario on the screen.

At the beginning of the object placing task, the subject kept both hands in the middle of own thighs, and was asked to move the virtual ball until it was placed inside a yellow cube (Fig. 2B) positioned at a forward and vertical distance of 36 and 26 cm, respectively, from the hand initial position.

At the beginning of the forearm pronation task, the subject kept the elbow angle at 90°, the wrist fully supinated and the shoulder laterally rotated so that the forearm was approximately 45° relative to the thigh. The subjects were then asked to move and rotate a virtual donut until it was placed inside a yellow cube (Fig. 2D) positioned at a medial and vertical distance of 52 cm and 12 cm, respectively, from the hand initial position. As the hand moved, the wrist pronated smoothly.

The experimental setup and markers’ protocol for providing kinematics of upper limb have been already published^11^ and briefly are reported below. For both tasks, only the forward movement of the hand towards the target was considered, and not the movement back to the initial resting position. For both tasks and sides, kinematics of upper limb and trunk were recorded using a 9-camera optoelectronic system (SMART209 DX, BTS, Italy) with a sampling frequency of 200 Hz. The system measured the 3D coordinates of nine spherical markers (10 mm diameter) attached to the following body landmarks: C7, manubrium, right and left acromions, lateral humeral condyle, ulnar and radial styloid processes, mid-forearm and hand of the tested limb. Markers’ coordinates were low-pass filtered at 6 Hz and then used to compute trunk, shoulder, elbow and wrist angles according to the joint coordinate system method^52^. Instants of initiation and termination of each movement were computed from the velocity of hand’s marker. In details, the beginning of the object placing (pronation) movement was identified with the first frame at which hand velocity exceeded a threshold of 5% of the maximum value, while its termination was the first frame at which hand velocity fell below the same threshold^11^. Hence, the time course of trunk and upper limb angles and marker trajectories were time normalized as a percentage of movement duration.

### Kinematic parameters for the assessment of motor performance

The following kinematic parameters were computed from each single trial and averaged across the repetitions for each participant at both T0 and T1:

- Amount of Elbow Extension (object placing task): computed as the elbow angle at the end of the task with respect to the elbow angle at the beginning of the movement in the sagittal plane. Lower negative values indicate larger amount of extension.
- Mean RMS of the angle of the trunk and shoulder joints (object placing task): computed as the average root-mean-square difference between the mean curve representing the joint angular movement in the sagittal plane of each participant post-stroke and the mean reference curve from the healthy subjects. Higher values indicate greater deviation from normal sagittal movement.
- Amount of Wrist Pronation (forearm pronation task): computed as the wrist maximum angle with respect to the wrist angle at the beginning of the movement in the horizontal plane. Higher positive values indicate larger amount of pronation.
- Mean RMS of the angle of the trunk and shoulder joints (forearm pronation task): computed as the average root-mean-square difference between the mean curve representing the joint angular movement in the horizontal (trunk) and frontal (shoulder) of each participant post-stroke and the mean reference curve from the healthy subjects.
- Movement smoothness (object placing and forearm pronation tasks) was assessed through the number of peaks of the velocity of the hand marker trajectory with respect to the shoulder marker one. Lower values indicate better smoothness^53^.

### Extraction of muscle synergies

Muscle activity was recorded with surface electrodes for electromyography (CometaWavePlus, Cometa Srl, Italy). Electrodes were placed according to guidelines of the Surface Electromyography for the Non-Invasive Assessment of Muscles European Community project – SENIAM^54^ and Anatomical guideline^55^. The activities of the following 16 muscles were recorded from each upper limb for reaching and pronation tasks: triceps brachii lateral (TBLH) and medial head (TBMH), biceps brachii short (BBSH) and long head (BBLH), anterior deltoid (ANDE), lateral deltoid (LADE), posterior deltoid (PODE), upper trapezius (UPTR), rhomboideus major (RHMA), brachioradialis (BRRA), supinator (SUPI), brachialis (BRAC), pronator teres (PRON), pectoralis minor (PEMI), infraspinatus (INFR) and teres major (TEMA).

Raw EMG signals were pre-processed in accordance with previous literature^56^. In order not to alter the variability in EMG, the signal of each muscle was amplitude-normalized to its peak value across all recorded trials. All data were time normalized to 100% of movement duration and subsequently averaged.

For each task, muscle synergies were extracted from the averaged EMG envelope of each subject using the Non Negative Matrix Factorization algorithm.

Briefly, for each subject, the EMGs were combined into an *m* × *t* matrix, where *m* indicates the number of muscles and *t* is the time base (t = trial × 101). Iteratively muscle synergies were extracted from 1 to 16, the number of muscles recorded.

The solution with a cross-validated EMG reconstruction factor R^2^ for > 90% was selected, thus obtaining two matrices for each extracted muscle synergy: an m × 1 array, which specifies the relative weighting of each muscle in the module (W, module composition) and a 1 × t array, which specifies the activation timing profile of the module (C).

### Muscle synergies parameters for the assessment of motor performance

The following muscle synergies parameters were calculated^56^:

- module similarity (W): maximum scalar product of the muscle weightings of each module between each post-stroke participant and HS group. Higher values indicate more similarity in module compositions.
- activation profile (C) similarity: Pearson's correlation coefficient of the activation profile of each module between each post-stroke participant and HS group. Higher correlations indicate more similarity in module compositions.

### Statistical analysis

Patients were grouped according to the treatment they received (between-Factor “Therapy”, Robot or Usual Care Group, RG or UCG).

The normality of data distribution and homogeneity of variances were assessed by Shapiro–Wilk and Levene test, respectively. Chi-square tests were used to compare sex, stroke type, paretic side, lesion location and chronicity. Mann–Whitney U Tests were used to compare time since stroke, while t-tests for independent samples (RG vs UCG) were used to compare baseline FM-UE score and muscle synergies parameters. Baseline kinematic parameters were compared using ANOVA (RG vs UCG vs HS). Post-hoc analysis (Fisher’s LSD test) was used to verify statistically significant differences among groups.

Pre to post Change-scores of the muscle synergies and kinematic parameters were compared between treatment groups following separate ANCOVA tests with baseline scores as covariates.

The significance level was set at *P* < 0.05, and values of *P* ranging from 0.05 and 0.1 included were considered as near-significant trend^57^.

### Sample size

As reported in our previous study^11^, the sample size was estimated on the basis of the kinematic outcome measure related to shoulder and elbow coordination. These data showed a post-training improvement in favor of the robot group with a Cohen’s d effect size of 1.40, which indicated that 24 subjects (12 per group) were necessary to obtain a difference between groups with α = 0.05 and Power (1 − β) = 0.9. In addition, the sample size of 15 or 17 in 1 group was considered appropriate for the randomized controlled trial at the demonstration-of-concept pilots, such as the present study^58^.

## Data Availability

The datasets generated during and/or analyzed during the current study are available from the corresponding author upon reasonable request.
